# Brand Awareness, Purchase Intention, and Branded Beef Purchasing Behavior: An Empirical Study Based on Jilin Province, China

**DOI:** 10.3390/foods15203645

**Published:** 2026-10-14

**Authors:** Xing-Long Yang, Yu-Wen Zhou, Kui-Xiu Teng, Yu-Shi Mao

**Affiliations:** 1 College of Economics and Management, Jilin Agricultural University, Changchun 130118, China; yangxinglong@jlau.edu.cn (X.-L.Y.); zhouyuwen@mails.jlau.edu.cn (Y.-W.Z.); 2 College of Biological and Agricultural Engineering, Jilin University, Changchun 130025, China; maoyushi0106@163.com

**Keywords:** branded beef, brand awareness, purchase intention, purchasing behavior, mediating effect

## Abstract

Against the backdrop of steadily growing beef consumption and accelerating competition toward brand differentiation, this study investigates how consumer brand awareness influences branded beef purchasing behavior. Drawing on survey data from 273 branded beef consumers in Jilin Province, China, we construct a theoretical framework linking brand awareness, purchase intention, and purchasing behavior, and empirically test it using binary logistic regression and probability-scale bootstrap mediation analysis, supplemented by KHB and LPM decompositions. Robustness checks include winsorized, Probit, and demographic-augmented specifications, and subgroup analyses use Firth’s penalized logistic regression where appropriate. The findings reveal that brand awareness is moderate with significant demographic heterogeneity; urban, married, household-shopper consumers exhibit higher awareness. Brand awareness exerts significantly positive effects on both purchase intention and purchasing behavior. Purchase intention partially mediates this relationship; based on the KHB decomposition and the linear probability model, the mediated share is approximately 11%, indicating that brand awareness primarily drives purchasing behavior directly. The positive effect is more pronounced among rural residents, females, middle-aged people, and middle-income groups. We recommend strengthening brand storytelling for the “Jilin Beef” regional brand, implementing precision marketing to enhance core channel penetration, and fostering government–enterprise collaboration to establish digital quality traceability, thereby supporting beef brand building and sustainable industry development.

## 1. Introduction

Agricultural brand building has emerged as a strategic lever for high-quality agricultural development in China. The 2025 No. 1 Central Document explicitly called for “cultivating premium agricultural brands,” while the 2026 document further mandated “accelerating standard upgrading and strengthening quality supervision and brand building,” continuously reinforcing the policy signal. Unlike ordinary agricultural products, branded agricultural products carry quality signals and value propositions, serving as a critical nexus linking quality upgrading on the production side with demand upgrading on the consumption side. In recent years, driven by rising household incomes and shifting dietary patterns, health consciousness, quality orientation, and safety considerations have become dominant logics in food consumption decisions. As a high-quality animal protein source, beef has witnessed steadily climbing consumer demand: per capita household purchases of beef and mutton in China more than doubled from 1.9 kg in 2000 to 3.9 kg in 2021 [1], and China is now the world’s second-largest beef consumer, with per capita consumption projected to rise by a further 0.8 kg (retail weight equivalent) by 2032 [2]. However, the production side exhibits a marked structural contradiction. Since 2023, persistently low beef market prices and frequent industry warning signals have rendered the competition model characterized by product homogenization unsustainable, accelerating the industry’s evolution toward a value-competition stage centered on brand differentiation [3].

Brands serve not only as essential tools for enterprises to convey quality information and capture premium pricing but also as critical heuristics for consumers to simplify decisions and reduce perceived risk in information-asymmetric markets [4]. Agricultural brand building represents an effective pathway to enhance competitiveness and realize value addition. International experience demonstrates that Japanese domestic beef has successfully withstood international low-cost competition by constructing a diversified value system encompassing quality, culture, and social function dimensions, along with corresponding price-formation mechanisms [5]. The key lies in effectively communicating the ecological, cultural, and social values behind the product to consumers through brand storytelling, thereby significantly enhancing willingness to pay and brand loyalty [6,7]. Therefore, strengthening brand building and enhancing consumer brand awareness holds significant importance for promoting the high-quality development of China’s beef industry. As a major beef cattle province, Jilin is vigorously advancing the “Jilin Beef” regional public brand, supported by the “Straw-to-Beef” ten-million-head cattle project and the Jizhi Jipin certification system. However, despite certifications obtained by enterprise brands such as “Haoyue” and “Benfu”, the overall market penetration and consumer awareness of branded beef remain considerably low. Against this backdrop, uncovering the mechanism through which consumer brand awareness drives purchasing behavior carries important practical significance for facilitating the industry’s transition from price-based to value-based competition.

How brand awareness influences consumer behavior represents a core issue shared by brand equity theory and the Theory of Planned Behavior (TPB). Keller’s brand equity theory posits that brand awareness—the representation and processing of consumer brand knowledge—constitutes the cornerstone of brand equity and can directly trigger purchase decisions [8]. In contrast, Ajzen’s TPB constructs a “cognition–intention–behavior” chain framework, emphasizing that behavioral intention is the most direct and powerful predictor of actual behavior [9,10]. Both theoretical streams have received extensive empirical support. In the agricultural product consumption domain, the positive effect of brand awareness on purchase intention has been widely validated across categories including green agricultural products [11,12], organic foods [13,14], and regional public brand products [15,16]. Xie and Hou [17] found that brand awareness influences purchase intention through the mediating roles of perceived quality, perceived value, and emotional perception, while Sun [18] confirmed that brand trust mediates the relationship between brand awareness and purchase intention. At the mechanistic level, Zou et al. [19] revealed that purchase intention plays a chain-mediating role between personal cognition and rural residents’ food safety consumption behavior, and studies by Bai et al. [20] and Gao et al. [21] on green agricultural product consumption confirmed that product cognition indirectly promotes purchasing behavior through mediators such as premium willingness and behavioral attitude.

Nevertheless, although existing research has confirmed the behavioral effects of brand awareness and the mediating role of purchase intention, the mechanism driving brand awareness’s influence on actual purchasing behavior remains insufficiently explored. TPB presupposes behavioral intention as the core mediator driving behavior, emphasizing that cognition acts on behavior indirectly via intention [9]. Yet, in mature consumption contexts, consumers may bypass elaborate psychological intention assessments and make purchase decisions directly based on brand awareness. Liu et al. [22] found that agricultural product brand image does not significantly affect purchase intention directly but influences behavior indirectly through brand loyalty, implying that brand awareness may affect behavior through multiple pathways whose relative weights vary by product characteristics and consumption context. In the unique consumption context of branded beef, where brands serve as core signals for simplifying decisions and mitigating risk, the purchase-driving mechanism may differ from that of general agricultural products; brand awareness might attenuate the role of purchase intention and directly trigger purchasing behavior. While the existing literature provides an important theoretical foundation, three gaps remain. First, regarding research context, existing studies predominantly focus on green agricultural products and organic foods, with relatively scarce attention to branded beef consumption scenarios. As a high-value fresh product, beef may exhibit distinctive branding pathways and consumer decision mechanisms. Second, regarding mechanism exploration, most studies stop at verifying the positive effect of awareness on behavior without adequately examining the intrinsic mechanism translating brand awareness into actual purchasing behavior. Whether brand awareness drives purchasing behavior primarily through direct effects or indirectly through purchase intention, and the relative magnitude of these effects, remain unclear. Third, regarding group heterogeneity, whether brand awareness’s impact on purchasing behavior differs significantly across consumer segments has received limited attention.

Accordingly, this study focuses on the branded beef market in Jilin Province, grounding its analysis in the Theory of Planned Behavior and brand equity theory. We construct a theoretical framework linking brand awareness, purchase intention, and purchasing behavior, and empirically test it using binary logistic regression (with winsorized, Probit, and demographic-augmented specifications as robustness checks), probability-scale bootstrap mediation analysis supplemented by KHB and LPM decompositions, and subgroup analyses using Firth’s penalized logistic regression where appropriate. The findings aim to provide theoretical foundations and decision-making references for the “Jilin Beef” regional brand building and enterprises’ precision marketing strategies. The potential marginal contributions of this study are twofold: (1) providing a more nuanced structural characterization of the “awareness–intention–behavior” mechanism in the specific consumption context of branded beef, and testing whether brand awareness can bypass intention to directly trigger behavior; and (2) revealing group differences in brand awareness effects through heterogeneity analysis, thereby providing empirical evidence for differentiated brand communication and precision marketing strategy formulation.

## 2. Theoretical Analysis and Research Hypotheses

### 2.1. Brand Awareness and Purchasing Behavior

Brand awareness refers to the process by which consumers receive, process, and memorize brand-related information, reflecting their ability to recognize a brand as belonging to a specific product category and to recall relevant brand knowledge; it constitutes the cornerstone of brand equity building [8]. In agricultural product markets, brand awareness serves critical functions of information transmission and quality signaling. According to brand equity theory, consumer brand knowledge comprises brand awareness and brand image; higher levels of brand awareness enable a brand to enter consumers’ consideration sets more readily and be prioritized during decision-making [23,24,25,26]. This effect is particularly pronounced in agricultural product markets characterized by high information asymmetry. Information asymmetry theory posits that when buyers and sellers possess significantly imbalanced information regarding product quality, brands act as “reputation collateral” that effectively transmits quality signals, thereby reducing consumer uncertainty and perceived risk [27]. Wang and Li [4] further confirmed that, under information-asymmetric conditions, brand awareness of safety-certified agricultural products significantly strengthens consumers’ purchase confidence.

From an information-processing perspective on consumer decision-making, brand awareness constitutes the core of consumption decisions. Zhuang and Yu [28] noted that brand awareness influences consumers’ attitude formation and purchase decisions through information-processing mechanisms. When consumers encounter branded beef products, the difficulty of directly observing intrinsic product quality renders the decision-simplifying function of brand awareness particularly critical. Jing [29] demonstrated that marketing supply quality encompassing brand and quality information significantly influences consumers’ purchasing behavior regarding specialty agricultural products in remote areas. Yang and Liu [30] further found that brand awareness not only significantly affects consumers’ current purchasing behavior but also indirectly influences future purchases through brand relationships. Based on the above analysis, this study proposes the following hypothesis:

**H1.**  *Brand awareness has a significant positive effect on branded beef purchasing behavior*.

### 2.2. Brand Awareness and Purchase Intention

Purchase intention refers to the psychological tendency and subjective probability of a consumer engaging in a specific purchasing behavior, reflecting the consumer’s subjective evaluation of and willingness to exert effort toward the purchase. The Theory of Planned Behavior (TPB) posits that behavioral attitude is a key antecedent of behavioral intention, with attitude originating from an individual’s beliefs about and evaluation of behavioral consequences [9,10]. Brand awareness shapes consumers’ overall evaluation of branded products, thereby influencing the formation of purchase intention. Specifically, positive awareness of brand quality, value, and safety can enhance consumers’ perceived value of and trust in the product, reduce perceived risk, and consequently strengthen purchase intention [31,32].

In the agricultural product consumption domain, the positive driving effect of brand awareness on purchase intention has been extensively validated. Xie and Hou [17] found that brand awareness influences purchase intention through multiple mediating pathways involving perceived quality, perceived value, and emotional perception. Zhang and Wei [33] confirmed that the origin image of agricultural products exerts a significant positive effect on consumers’ purchase intention. Sun [18] revealed that black rice brand awareness indirectly promotes purchase intention through the mediating role of brand trust. Regarding regional public brands, studies by Sun et al. [16] and Hu et al. [15] both demonstrated that awareness of regional public brands positively influences consumers’ purchase intention. Furthermore, in subcategories such as green agricultural products [11,12] and organic foods [13,14], awareness has been consistently identified as an important antecedent of purchase intention. Based on the above analysis, this study proposes the following hypothesis:

**H2.**  *Brand awareness has a significant positive effect on branded beef purchase intention.*

### 2.3. Purchase Intention and Purchasing Behavior

The core proposition of the TPB is that behavioral intention is the most direct and powerful predictor of actual behavior [9]. Numerous studies support the significant predictive power of behavioral intention on actual behavior, and Notani [10] further identified perceived behavioral control as a moderator of the intention–behavior relationship. Purchase intention reflects consumers’ readiness state and psychological commitment to implementing a purchase, serving as a critical bridge connecting internal cognitive evaluation with external market behavior.

Although an “intention–behavior gap” exists in reality, the significant predictive power of purchase intention on purchasing behavior has been validated across multiple food consumption domains. In the green agricultural product field, Guo et al. [12] found that purchase intention is an important antecedent driving actual consumption behavior. In the organic food domain, Wei and Hu [14] verified the positive effect of purchase intention on purchasing behavior. In the context of consumption-assisted products, Sun and Bu [34] also found that purchase intention significantly predicts consumption assistance behavior. Apdillah et al. [35] similarly validated the positive impact of purchase intention on purchase decisions. Based on the above analysis, this study proposes the following hypothesis:

**H3.**  *Purchase intention has a significant positive effect on branded beef purchasing behavior.*

### 2.4. The Mechanism Linking Brand Awareness, Purchase Intention, and Purchasing Behavior

Brand awareness, purchase intention, and purchasing behavior constitute a “cognition–intention–behavior” chain mechanism, consistent with the hierarchical logic of “cognition–affect–behavior” in cognitive psychology. That is, consumers first form cognitive evaluations of a brand, subsequently generate affective tendencies and behavioral intentions, and ultimately drive actual behavior. This chain mechanism has been validated from multiple perspectives in agricultural product consumption research. Zou et al. [19] found that purchase intention plays a significant positive chain-mediating role between personal cognition and rural residents’ food safety consumption behavior. Bai et al. [20] and Gao et al. [21] confirmed that, in green agricultural product consumption, product cognition indirectly promotes purchasing behavior through mediators such as behavioral attitude and premium willingness. Sun and Bu [34] found that ecological value cognition influences purchase intention for consumption-assisted products through multiple mediators including attitude, subjective norms, and perceived behavioral control, with attitude exhibiting the strongest mediating effect.

Regarding indirect effects, Ajzen explicitly stated that behavioral intention is the most immediate antecedent of behavior, with behavioral attitude, subjective norms, and perceived behavioral control all acting indirectly on behavior through intention [9]. As an important determinant of behavioral attitude, brand awareness should primarily transmit its influence on purchasing behavior through purchase intention. In the branded beef consumption context, the more positive consumers’ quality awareness of a brand, the more readily they form purchase intention and subsequently convert it into actual purchases.

Regarding direct effects, Keller’s brand equity theory emphasizes that strong brand knowledge can directly constitute a consumer purchase heuristic, reducing information search costs and decision complexity [8]. In credence goods markets characterized by high information asymmetry, brands serve as “reputation signals” with stronger direct decision-triggering functions. Jiang and Zhu [27] proposed a brand effect mechanism transcending the “cognition-attribute” paradigm, noting that brand awareness can directly drive behavior through holistic evaluation without necessarily undergoing elaborate attribute trade-offs and intention assessments. In the branded beef consumption context, consumers face high information asymmetry regarding product quality, and purchase decisions involve considerable economic costs and safety risks. Consequently, established brand awareness is more likely to constitute a “decision heuristic,” whereby consumers bypass elaborate intention assessments and make purchase choices directly based on brand trust. Liu et al. [22] provided indirect evidence for this logic: agricultural product brand image did not significantly affect purchase intention directly but influenced behavior indirectly through brand loyalty, implying that the pathways through which brand awareness affects behavior may vary by product characteristics and consumption context. Based on the above theoretical analysis, this study proposes the following hypothesis:

**H4.**  *Purchase intention mediates the effect of brand awareness on purchasing behavior.*

## 3. Materials and Methods

### 3.1. Data Source

The data used in this study were collected through a questionnaire survey conducted by a research team in four cities of Jilin Province, China—Changchun, Jiaohe, Baicheng, and Yanji—from August to September 2025. A venue-based intercept (convenience) sampling approach was adopted. Trained interviewers approached beef consumers on-site at large supermarkets and meat specialty stores in the four cities and invited them to complete the questionnaire. Although this procedure does not constitute probability sampling from a defined sampling frame, it ensured that all respondents were actual beef consumers encountered in real purchase settings. This sampling strategy limits the generalizability of the findings, as further discussed in the Limitations subsection. A total of 300 questionnaires were distributed and collected. After excluding responses with unreasonable answers or missing key items, 273 valid questionnaires were retained, yielding a valid response rate of 91.0%.

### 3.2. Variable Measurement and Reliability and Validity

#### 3.2.1. Variable Measurement

Questionnaire development. The questionnaire was developed based on Keller’s brand equity theory and Ajzen’s Theory of Planned Behavior, and item wording was adapted from established instruments in the brand awareness, purchase intention, and agricultural product consumption literature. The research team reviewed all items for clarity, relevance, and consistency with the theoretical constructs. A formal pilot survey was not conducted before the main survey because of time and resource constraints; this limitation is acknowledged in the Limitations Subsection. Mesinger et al. [36] provide a useful methodological reference for scale development and validation. The final questionnaire covered brand awareness, purchase intention, product attribute importance, purchasing behavior, and demographic characteristics.

The dependent variable in this study was branded beef purchasing behavior (PB), measured by the item “Have you ever purchased branded beef?” Responses were coded as 1 for “Yes” and 0 for “No,” constituting a binary variable.

The core independent variable was brand awareness (BA). Brand awareness captures the extent to which a brand is present in consumers’ memory, and self-rated familiarity is a well-established operationalization of this construct [8]; self-assessed measures of product knowledge have been shown to be valid and to predict behavior independently of objective knowledge [37]. Following Bergkvist and Rossiter [38], who demonstrated that single-item measures are appropriate when a construct consists of a concrete singular object and a concrete attribute, BA was measured with a single item: “How familiar are you with branded beef?” (Not at all familiar = 1; slightly familiar = 2; moderately familiar = 3; fairly familiar = 4; very familiar = 5).

The mediating variable was purchase intention (PI). Based on the behavioral intention measurement paradigm of the Theory of Planned Behavior (TPB) [9] and the specific context of branded beef consumption, PI was measured with four items covering post-understanding intention (PI1), certification label preference (PI2), brand extension willingness (PI3), and quality control satisfaction (PI4). Each item was rated on a 5-point Likert scale, and PI was computed as the mean of the four items.

To control for the potential influence of product attributes on purchasing behavior, seven core product attribute factors were included as control variables: price, quality, taste, safety, packaging, nutritional value, and cattle breed. Previous studies have shown that these product attributes are important factors influencing consumers’ purchasing decisions for fresh food products [29,33]; controlling for these variables helps to more accurately identify the independent effect of brand awareness. All product attribute variables were measured using a 5-point Likert importance scale. Variable definitions and descriptive statistics are presented in Table 1.

#### 3.2.2. Reliability and Validity Testing

Prior to the reliability assessment, the purchase intention scale was purified based on the survey data. Exploratory factor analysis of the six original intention items indicated that the four retained items loaded on a single factor (loadings 0.684–0.816), whereas the remaining two items loaded on a separate factor. Retaining the four items improved Cronbach’s alpha from 0.699 to 0.778; the two items were therefore excluded, and the retained items were renumbered PI1–PI4.

**Figure 1:**
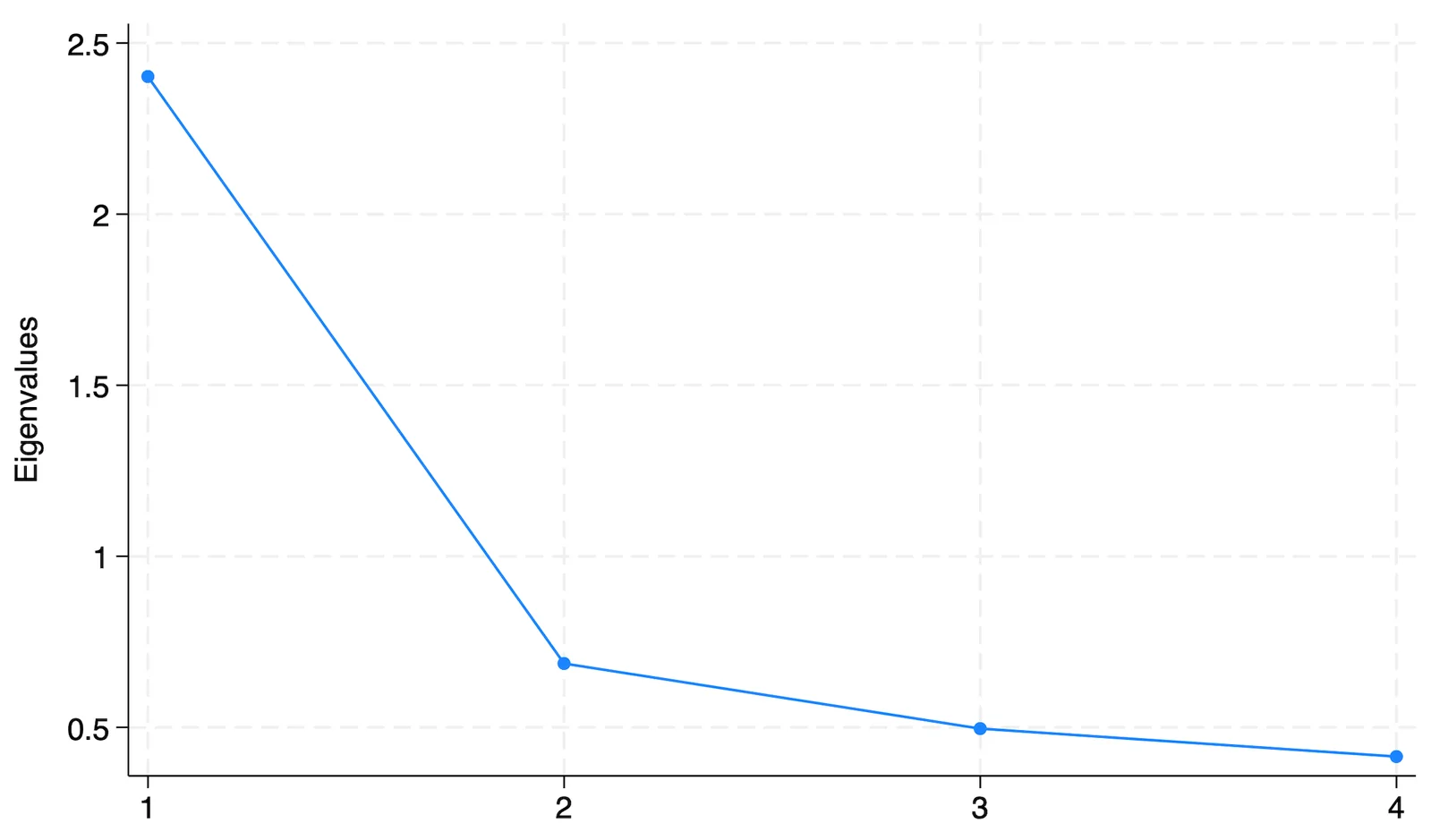
Scree plot of the principal component analysis on the four purchase intention items. The first factor (eigenvalue = 2.402) clearly dominates; all remaining eigenvalues are below 1.

Reliability and validity were assessed for the four-item purchase intention (PI) scale. Brand awareness was measured with a single item; therefore, Cronbach’s α is not applicable. The PI scale showed satisfactory internal consistency, with Cronbach’s α = 0.778. Table 2 reports the item–total correlations and the α coefficient if each item was deleted; all values remained within an acceptable range, indicating that no single item disproportionately weakened the scale. Exploratory factor analysis (EFA) using principal component analysis (PCA) was then conducted on the four retained PI items. For these four items, the Kaiser–Meyer–Olkin (KMO) measure of sampling adequacy was 0.763, and Bartlett’s test of sphericity was significant (χ^2^(6) = 292.60, *p* < 0.001), confirming that the items were suitable for factor analysis. PCA extracted a single factor with an eigenvalue of 2.402, accounting for 60.06% of the total variance. All four items loaded strongly on this factor (see Table 3). The scree plot in Figure 1 shows a clear break after the first factor, supporting the retention of a single factor. These results indicate that the PI scale has good structural validity.

#### 3.2.3. Common Method Bias Test

**Figure 2:**
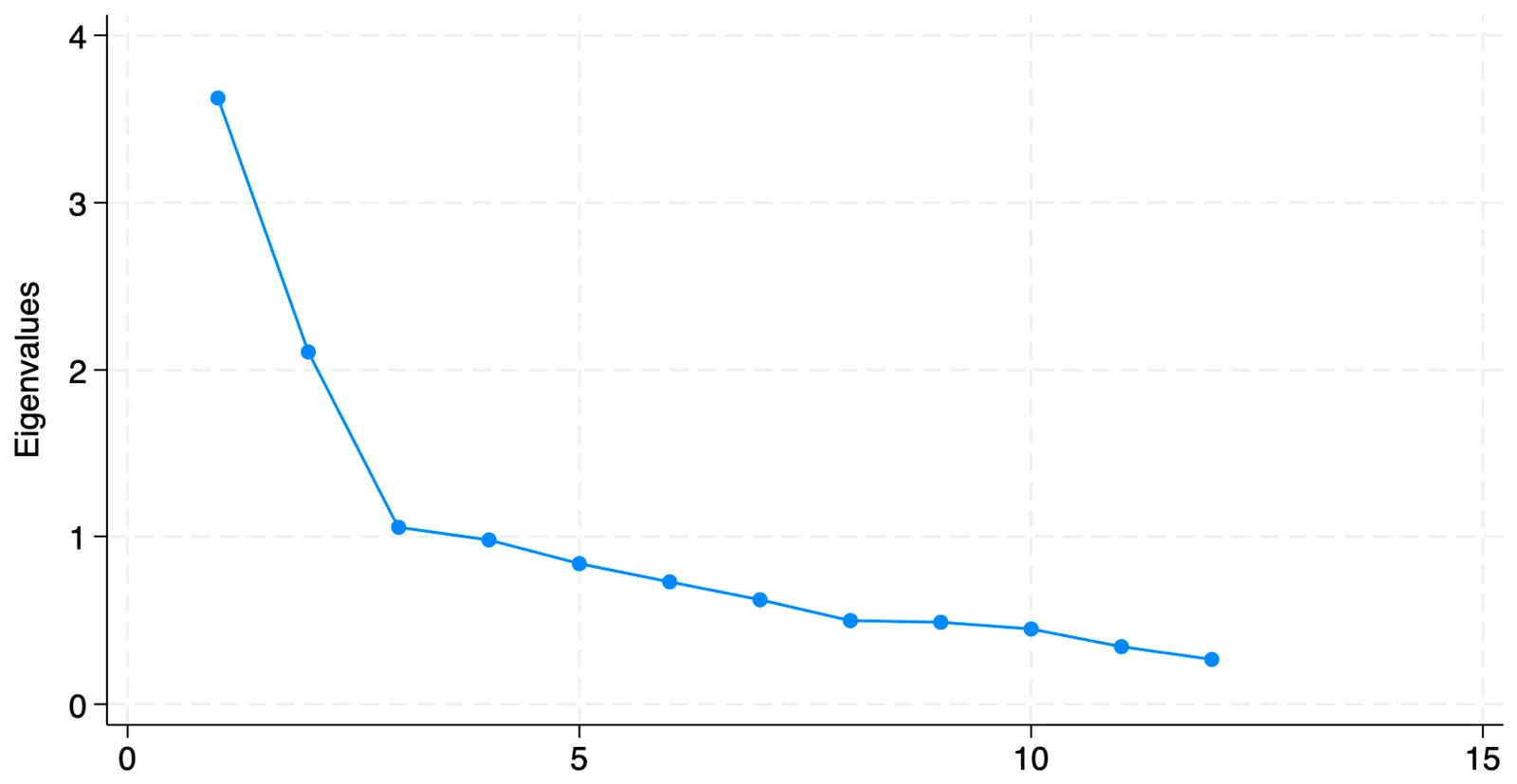
Scree plot of the unrotated principal component analysis on all 12 measurement items. Three factors have eigenvalues greater than 1, and the first factor explains 30.21% of the variance, below the 40% threshold.

Given that all data were collected via self-reported questionnaires, common method bias (CMB) may be a concern. At the procedural level, anonymous completion was guaranteed, and respondents were explicitly informed that there were no right or wrong answers. At the statistical level, Harman’s single-factor test was employed. An unrotated principal component analysis of all 12 measurement items (BA, PI1–PI4, and the seven product attributes) extracted three factors with eigenvalues greater than 1. The first factor explained 30.21% of the total variance, below the critical threshold of 40%; the three factors collectively explained 56.56% of the total variance. The corresponding scree plot is presented in Figure 2. Both procedural and statistical tests suggest that common method bias was not a serious problem in this study, and the data quality was adequate for subsequent hypothesis testing.

#### 3.2.4. Multicollinearity Test

To avoid estimation bias caused by high correlations among explanatory variables, a multicollinearity diagnosis was performed prior to regression analysis using variance inflation factors (VIF) and tolerance as diagnostic indicators. As shown in Table 4, all VIF values ranged from 1.02 to 2.45, with a mean VIF of 1.56, well below the critical threshold of 10, and tolerance values ranged from 0.409 to 0.976, all above the minimum standard of 0.1. These results indicate that severe multicollinearity was not present among the explanatory variables, and the coefficient estimates of the regression models were stable and reliable for subsequent analysis.

### 3.3. Model Specification

#### 3.3.1. Binary Logistic Regression Model

To examine the effect of brand awareness on purchasing behavior, a binary logistic regression model was constructed. Since the dependent variable PB is binary (1 = purchased, 0 = not purchased), the binary logistic model was employed for estimation, as specified in Equation (1):

(1) $$\mathrm{Logit}\left(P\left(\mathrm{PB}=1\right)\right)=\alpha_{0}+\alpha_{1}\mathrm{BA}+\sum \delta_{i}{\mathrm{Controls}}_{i}+\varepsilon_{1}$$

where PB denotes branded beef purchasing behavior; Logit(P(PB = 1)) is the log-odds of PB equaling 1; BA denotes brand awareness; Controls_i_ represents the control variables (price, quality, taste, safety, packaging, nutritional value, and cattle breed); α_0_ is the constant term; and ε_1_ is the random error term.

#### 3.3.2. Mediation Effect Test Model

To test the mediating role of purchase intention in the relationship between brand awareness and purchasing behavior, we first specify the mediation equations. Because purchasing behavior (PB) is binary, the indirect effect cannot be computed by simply multiplying the OLS coefficient of the BA → PI path with the logit coefficient of the PI → PB path: the two coefficients are on different scales (probability scale vs. log-odds scale), and the exact identity “direct effect + indirect effect = total effect” does not hold when one path uses a nonlinear link function. We therefore adopt a three-pronged strategy that keeps all effect decompositions on a common scale.

First, following Hayes and Preacher [39], we compute the indirect effect on the probability scale as the product of (a) the OLS coefficient of BA in the PI equation and (b) the average marginal effect of PI on the purchase probability obtained from the logit model, and test it with 5000 bootstrap resamples (percentile and bias-corrected confidence intervals are reported).

Second, as a unified-scale cross-check, we re-estimate all three equations as linear probability models (LPMs), in which the conventional product-of-coefficients decomposition is exact, again with 5000 bootstrap resamples.

Third, we apply the KHB decomposition (Karlson, Holm and Breen [40]), a method specifically developed to decompose total effects into direct and indirect components in nonlinear probability models, which additionally provides analytic standard errors.

The probability-scale bootstrap approach is treated as the primary method; the LPM and KHB decompositions serve as cross-validations of the mediated share.

The mediation effect models are specified in Equations (2) and (3):

(2) $$\mathrm{PI}=\alpha_{0}+\alpha_{1}\mathrm{BA}+\sum \delta_{i}{\mathrm{Controls}}_{i}+\varepsilon_{2}$$

(3) $$\mathrm{Logit}\left(P\left(\mathrm{PB}=1\right)\right)=\alpha_{0}+\alpha_{1}\mathrm{BA}+\alpha_{2}\mathrm{PI}+\sum \delta_{i}{\mathrm{Controls}}_{i}+\varepsilon_{3}$$

where PB denotes branded beef purchasing behavior; Logit(P(PB = 1)) is the log-odds of PB equaling 1; BA denotes brand awareness; PI denotes purchase intention; Controls_i_ represents the control variables (price, quality, taste, safety, packaging, nutritional value, and cattle breed); α_0_ is the constant term; and ε_i_ represents the random error terms.

All statistical analyses in this study were performed using Stata 18.0 (StataCorp LLC, College Station, TX, USA).

## 4. Results

### 4.1. Descriptive Statistical Analysis

Prior to group comparisons, we examined whether the assumptions for parametric tests were met. Normality was checked using the Shapiro–Wilk test (W = 0.998, *p* = 0.997), and homogeneity of variance was checked using Levene’s test, which indicated unequal variances for household income (*p* < 0.001). Because brand awareness was measured on a single 5-point ordinal item and the group sizes were highly unbalanced, the assumptions for parametric tests were not fully met. Therefore, non-parametric tests were used for group comparisons: Mann–Whitney U tests for binary groups and Kruskal–Wallis tests for multi-category groups. Given the unbalanced group sizes, these results are interpreted descriptively rather than as robust evidence of population differences.

The analysis of brand awareness levels across demographic groups is presented in Table 5. Overall, the mean brand awareness score among the surveyed Jilin beef consumers was 2.88, indicating a moderate level with considerable room for improvement. Regarding place of residence, urban consumers exhibited significantly higher brand awareness than rural consumers (Mann–Whitney z = −2.45, *p* = 0.014), reflecting a marked imbalance in brand marketing and communication channels between urban and rural areas and the relative inadequacy of brand penetration in rural regions. Regarding family role, married consumers and those responsible for household grocery shopping showed significantly higher brand awareness than their counterparts (married: z = −2.22, *p* = 0.027; household buyer: z = −3.01, *p* = 0.003), possibly because family responsibility roles strengthen consumers’ attention to and learning of brand information. Regarding education, no statistically significant differences in brand awareness were observed across educational levels (Kruskal–Wallis χ^2^ = 6.41, *p* = 0.170). Regarding gender, male consumers showed slightly higher brand awareness than females (means of 3.03 vs. 2.82), but the difference was not statistically significant (Mann–Whitney z = 1.73, *p* = 0.084). Regarding age, consumers aged 36–45 and over 55 showed relatively higher brand awareness, whereas those aged 18–25 showed the lowest level; however, the overall between-group difference did not reach significance at the 5% level (Kruskal–Wallis χ^2^ = 8.32, *p* = 0.080). Regarding household income, the Kruskal–Wallis test indicated a significant difference at the 5% level (χ^2^ = 10.67, *p* = 0.031), but given the highly unbalanced group sizes, this result should be interpreted with caution.

### 4.2. Baseline Model Regression

To investigate the effect of brand awareness on branded beef purchasing behavior among Jilin consumers, a binary logistic regression model was constructed. Model 1 included only the seven product attribute variables (price, quality, taste, safety, packaging, nutritional value, and cattle breed) as controls. Model 2 added brand awareness to Model 1 to test its independent effect after controlling for product attributes. The baseline regression results are reported in Table 6.

The likelihood ratio chi-square value of Model 1 was 19.04, with Cox & Snell R^2^ and Nagelkerke R^2^ of 0.067 and 0.098, respectively. The likelihood ratio chi-square value of Model 2 increased to 53.50, with Cox & Snell R^2^ and Nagelkerke R^2^ rising to 0.178 and 0.260, respectively. These results indicate that the model fit improved after the inclusion of brand awareness.

In Model 1, price, nutritional value, and cattle breed significantly influenced purchasing behavior at the 5% level. Price exerted a significant negative effect, indicating that higher branded beef prices reduce the likelihood of purchase. Nutritional value also showed a significant negative effect, possibly because consumers associate high nutritional value with high prices, generating psychological budget constraints. Cattle breed exhibited a significant positive effect, suggesting that consumers hold certain preferences for premium cattle breeds and that breed awareness is an important driver of branded beef choice.

In Model 2, brand awareness significantly and positively influenced purchasing behavior at the 1% level, with a coefficient of 1.064 and an odds ratio (Exp(B)) of 2.90. This indicates that for every one-unit increase in brand awareness, the odds of consumers purchasing branded beef increase by approximately 2.90 times, thereby supporting H1. Notably, nutritional value and cattle breed, which were significant at the 5% level in Model 1, weakened in significance in Model 2, remaining significant only at the 10% level. This suggests that brand awareness partially substitutes for consumers’ independent evaluation of specific product attributes. From the perspective of brand equity theory, brands serve as comprehensive quality signals that simplify consumers’ decision pathways, making them rely more on holistic brand awareness than on single-attribute judgments [8].

The fitted Model 2 equation is

Logit(P(PB = 1)) = −1.602 + 1.064·BA − 0.327·Price + 0.246·Quality + 0.136·Taste + 0.206·Safety − 0.205·Packaging − 0.446·Nutritional value + 0.294·Cattle breed

That is, each one-unit increase in brand awareness multiplies the odds of purchasing branded beef by e^1.064^ ≈ 2.90, holding product attributes constant.

### 4.3. Mediation Effect Test

To test the mediating role of purchase intention, the probability-scale bootstrap approach described in Section 3.3.2 was used as the primary method. The results are reported in Table 7 and Table 8.

Model 3 in Table 7 tests the effect of brand awareness on purchase intention. The results show that the coefficient of brand awareness was 0.134, significant at the 1% level, indicating that brand awareness has a significant positive effect on purchase intention; thus, H2 is supported. Model 4 further tests the effects of brand awareness and purchase intention on purchasing behavior. The results show that the coefficient of purchase intention was 0.978, significant at the 1% level, indicating that purchase intention significantly and positively affects purchasing behavior; thus, H3 is supported. Brand awareness also remained significant at the 1% level, with a coefficient of 1.034.

The fitted equations are

$$\mathrm{PI}=2.732+0.134\cdot \mathrm{BA}+\sum \delta_{i}{\mathrm{Controls}}_{i}$$

$$\mathrm{Logit}\left(P\left(\mathrm{PB}=1\right)\right)=-4.330+1.034\cdot \mathrm{BA}+0.978\cdot \mathrm{PI}+\sum \delta_{i}{\mathrm{Controls}}_{i}$$

The mediation effect test results in Table 8 show that the direct effect of brand awareness on purchasing behavior was 0.165, with a 95% confidence interval excluding zero, indicating a significant direct effect. The indirect effect through purchase intention was 0.021, with a 95% confidence interval also excluding zero, indicating a significant indirect effect. These results indicate that purchase intention plays a partial mediating role between brand awareness and purchasing behavior; thus, H4 is supported.

Effect decomposition. Because the outcome model is nonlinear, the direct and indirect effects on the probability scale are not exactly additive. To obtain an interpretable share of the total effect, the KHB decomposition and the LPM decomposition were applied. As shown in Table 9, the KHB decomposition indicates that the indirect effect accounted for 11.2% of the total effect and the direct effect accounted for 88.8%, while the LPM decomposition yields a consistent mediated share of 11.3% (direct effect: 88.7%). From the perspective of brand equity theory, the direct pathway predominated in the branded beef consumption context. This implies that consumers with high brand awareness may not need to undergo the “awareness–intention–behavior” cognitive processing sequence; instead, they can make purchase decisions directly based on established brand trust. Strong brand awareness may thus simplify the decision process, shifting consumers from a “high-intention-driven” to a “high-awareness-driven” decision mode.

### 4.4. Robustness Checks

To verify the reliability of the baseline regression results, robustness checks were conducted along three dimensions. First, continuous variables were winsorized at the 5th and 95th percentiles to eliminate potential interference from extreme values. Second, a Probit model was used in place of the logistic model to test the sensitivity of the findings to model specification. Third, demographic variables including gender, age, education, residence, marital status, household income, and household buyer were added to the original control variables to address possible omitted-variable bias. The results are reported in Table 10. In Models 5, 6, and 7, the coefficients for brand awareness were all positive and significant at the 1% level. Across all three specifications, the direction, significance, and magnitude of the core explanatory variable remained materially unchanged, confirming the robustness of the baseline regression conclusions.

### 4.5. Heterogeneity Analysis

To explore group differences in the effect of brand awareness on purchasing behavior, subgroup logistic regressions were conducted across four dimensions: gender, residence, age, and household income level. For subgroups with small samples or indications of separation, Firth’s penalized logistic regression was used to obtain more reliable estimates. The estimator used in each column and the results are reported in Table 11.

Gender. The positive effect of brand awareness on purchasing behavior was significant among female consumers but not among male consumers. Although males exhibited slightly higher brand awareness levels than females in the descriptive analysis, females demonstrated a stronger conversion effect from awareness to behavior. One possible interpretation is that, consistent with the frequently observed pattern in which women take primary responsibility for household food purchasing, female respondents may convert brand awareness into purchase behavior more efficiently. Given the smaller male subsample, this gender difference should be regarded as exploratory.

Residence. The coefficient of brand awareness for rural consumers was substantially higher than that for urban consumers. Descriptive statistics showed that rural consumers had significantly lower brand awareness than urban consumers; however, the heterogeneity analysis suggests that their conversion efficiency from awareness to behavior may be higher. One possible interpretation is that brand information may be relatively scarce in rural markets. Once rural consumers form brand awareness, the limited availability of information substitutes may mean that brand trust is more readily converted into purchasing behavior. Because the rural subsample is small, this result should be interpreted with caution.

Age. The coefficient of brand awareness was highest among the middle-aged group (36–45 years), followed by the older group (>45 years), and lowest among the young group (18–35 years). Middle-aged consumers may have more stable income foundations, greater influence over household consumption decisions, and higher food safety concerns, which may give brand awareness greater weight in their purchase decisions.

Household income. The effect of brand awareness was strongest among the middle-income group (CNY 5001–10,000), followed by the low-income group (≤CNY 5000) and the high-income group (>CNY 10,000). The middle-income group may possess certain purchasing power while remaining price-sensitive; brand awareness may therefore play a more important role in their quality screening and risk-aversion decisions. For the high-income group, brand consumption may already be habitual, leading to a diminishing marginal effect of brand awareness. For the low-income group, stronger budget constraints may inhibit the conversion from awareness to behavior to some extent.

## 5. Discussion

This study yields findings that speak to the ongoing debate between brand equity theory and the Theory of Planned Behavior regarding how cognition translates into consumption behavior. Three aspects of the results warrant discussion.

First, the dominant direct pathway from brand awareness to purchasing behavior challenges the TPB presumption that cognition must act on behavior through intention. In our sample, purchase intention transmitted only about 11% of the total effect of brand awareness on branded beef purchasing behavior, with both the KHB decomposition for nonlinear models and the linear probability model yielding consistent estimates. This pattern is consistent with Keller’s argument that strong brand knowledge can function as a purchase heuristic, and with the “cognition-driven” decision mode observed in mature branded food categories: once brand trust is established, consumers may bypass elaborate intention formation and decide directly. For credence goods such as branded beef, where intrinsic quality is difficult to verify at the point of purchase, a familiar brand name may itself serve as a sufficient quality signal, which would explain why the direct effect is so much larger than the indirect effect through intention.

Second, the finding that the awareness-to-behavior conversion is stronger among rural consumers, despite their significantly lower average brand awareness, suggests an asymmetry between brand communication and brand conversion. In rural markets, fewer information substitutes appear to make established brand awareness a scarcer and therefore more decisive signal. However, given the small rural subsample, this interpretation is speculative and requires verification with larger samples.

Third, the subgroup patterns across gender, age, and income suggest that the association between brand awareness and purchasing behavior may not be uniform across consumer segments. In our sample, the association was relatively stronger among middle-aged, middle-income, and female consumers. One possible interpretation is that these groups may be more involved in household food purchasing, although this mechanism was not directly tested in the present study. These patterns tentatively suggest that precision marketing aimed at core household purchasers may be more cost-effective than broad awareness campaigns. However, given the small and unbalanced subgroup samples, these implications should be regarded as exploratory and require confirmation in larger, more balanced samples.

These findings should be interpreted within the limits of the study. The cross-sectional design precludes causal claims; the convenience sample constrains generalizability; and the small size of several subgroups renders the heterogeneity results exploratory. Nevertheless, the convergence of the parametric, non-parametric, and penalized estimation results strengthens confidence in the central conclusion that brand awareness exerts a robust positive effect on branded beef purchasing behavior, primarily through a direct pathway.

## 6. Conclusions and Implications

Drawing on brand equity theory and the Theory of Planned Behavior (TPB), this study systematically examined the mechanism through which brand awareness influences branded beef purchasing behavior, using survey data from 273 consumers in Jilin Province, China. Binary logistic regression, probability-scale bootstrap mediation analysis, effect decomposition, and heterogeneity analysis were employed. The main findings are as follows.

First, brand awareness among the surveyed Jilin beef consumers is moderate, with considerable room for improvement. Significant differences exist across demographic groups; urban, married, and household grocery shoppers exhibit relatively higher awareness, while age differences approach significance, reflecting group-based asymmetries in brand communication and market penetration.

Second, in our sample, brand awareness exerts a significant positive effect on both purchase intention and purchasing behavior, and its direct effect on purchasing behavior is robust to winsorization, Probit specification, and the inclusion of demographic controls. This is consistent with the core role of brands as quality signals in agricultural product consumption decisions.

Third, purchase intention partially mediates the relationship between brand awareness and purchasing behavior. Because the outcome model is nonlinear, the mediated share was quantified with the KHB decomposition and cross-validated with the linear probability model; both indicate that the indirect effect accounts for approximately 11% of the total effect, whereas the direct effect accounts for about 89%. This suggests that, in our sample, consumers tend to rely on established brand trust and cognitive simplification when making branded beef purchase decisions, exhibiting a more “awareness-driven” than “intention-driven” decision characteristic.

Fourth, heterogeneity analysis reveals group differences within our sample; the positive effect of brand awareness is particularly pronounced among females, rural residents, middle-aged individuals, and middle-income groups. Given the unequal and, in some cases, small subgroup sizes, these patterns should be regarded as exploratory.

Based on these findings, we derive the following managerial and policy implications.

Brand Building. Enterprises should deepen brand storytelling by leveraging digital marketing, particularly e-commerce livestreaming and social media campaigns. By integrating Jilin’s cattle industry resources and cultural heritage, and emphasizing attributes such as “ecological ranches,” “green forage,” and “full-process traceability,” a compelling regional public brand narrative for “Jilin Beef” (Ji Niu) can be constructed. Immersive, experiential, and interactive scenarios should be employed to enhance consumer perceptions of brand safety and premium quality.

Precision Marketing. Differentiated strategies should be tailored to distinct consumer segments. For young potential consumers, social seeding and scenario-based marketing via platforms such as Bilibili and Douyin (TikTok), in collaboration with key opinion leaders (KOLs), can embed the brand in diverse consumption contexts and project a youthful brand image. For middle-income, quality-oriented consumers, premium customization and membership services—including exclusive delivery, custom cuts, and loyalty point systems—can enhance repurchase stickiness. For low-income, price-sensitive consumers, community group buying and channel penetration into lower-tier markets via platforms such as Meituan Youxuan and Duoduo Maicai can be utilized, with dedicated value zones designed to reduce intermediary costs.

Channel Penetration. Offline “Jilin Beef” brand experience zones should be established in supermarkets and meat specialty stores, where traceability QR codes and visualized meat quality inspection reports can enhance consumer trust. Online, short videos showcasing ranch forage, slaughtering, and processing should be distributed via short-video platforms to demonstrate product quality and safety. Collaborations with local KOLs for “factory livestreams” can further reinforce the “safe and traceable” brand image.

Policy Support. The Jilin provincial government should expedite the refinement of the Jizhi Jipin beef certification standards, establish a full-industrial-chain traceability system, and strengthen regional brand certification and oversight. The “Straw-to-Beef” initiative should be deeply integrated with brand building, and an “enterprise + cooperative + farmer” interest-linkage mechanism should be established to secure high-quality raw material supplies, ensure beef product quality and safety, and ultimately enhance consumer trust in quality.

Limitations. Several limitations of this study should be acknowledged. First, the data were collected through venue-based intercept convenience sampling in four cities of Jilin Province; therefore, the findings describe the surveyed sample and should be interpreted with caution when generalized beyond the study sample. Second, the cross-sectional design does not allow strong causal inference, and the single-item measure of brand awareness, although justified by established measurement theory, cannot capture the full multidimensionality of the construct. Third, no formal pilot survey was conducted before the main survey, and the purchasing behavior measure relied on self-reported past purchases rather than observed behavior, which may introduce recall and social desirability bias. Fourth, several subgroups in the heterogeneity analysis were small, and their results are exploratory. Future research could employ probability sampling across multiple provinces, longitudinal or experimental designs, and objective behavioral or scanner data. In addition, future studies should develop and validate a multi-item brand awareness scale, conduct a formal pilot survey before the main survey, and use larger and more balanced subgroup samples to confirm the heterogeneity findings.

## Figures and Tables

**Table 1:** Variable definitions and descriptive statistics.

Variable	Definition and Coding	Mean	Std.Dev.
Purchasing behavior (PB)	Have you ever purchased branded beef? Yes = 1; No = 0	0.74	0.441
Purchase intention (PI)	Mean of PI1–PI4 (four items)	3.64	0.583
PI1	After fully understanding branded beef, you would choose to purchase it: strongly disagree = 1; disagree = 2; neutral = 3; agree = 4; strongly agree = 5	3.60	0.886
PI2	You would choose to purchase branded beef products that are certified and labeled with relevant marks: same as above	3.68	0.919
PI3	When you are satisfied with a branded beef product, you would try other beef products from the same brand: same as above	3.72	0.839
PI4	Satisfaction with the quality control effectiveness of branded beef enterprises: very dissatisfied = 1; dissatisfied = 2; neutral = 3; satisfied = 4; very satisfied = 5	3.51	0.832
Brand awareness (BA)	How familiar are you with branded beef? Not at all familiar = 1; slightly familiar = 2; moderately familiar = 3; fairly familiar = 4; very familiar = 5	2.88	0.895
Price	Price: not at all influential = 1; slightly influential = 2; neutral = 3; moderately influential = 4; very influential = 5	3.45	1.218
Quality	Quality: same as above	3.86	0.930
Taste	Taste: same as above	3.86	0.974
Safety	Safety: same as above	3.89	0.935
Packaging	Packaging: same as above	2.75	1.134
Nutritional value	Nutritional value: same as above	3.78	0.968
Beef cattle breed	Beef cattle breed: same as above	3.23	1.190

**Table 2:** Reliability analysis of the purchase intention scale (PI1–PI4).

Item	Item–Total Correlation	α If Item Deleted
PI1	0.777	0.725
PI2	0.835	0.675
PI3	0.714	0.764
PI4	0.768	0.724
Scale α	—	0.778

**Table 3:** Factor loadings of the purchase intention items.

Item	Factor Loading	Uniqueness
PI1	0.776	0.398
PI2	0.838	0.297
PI3	0.703	0.506
PI4	0.777	0.396
Eigenvalue	2.402	—
Variance explained	60.06%	—

**Table 4:** Multicollinearity test results.

Variable	Tolerance	VIF
Brand awareness	0.921	1.09
Purchase intention	0.892	1.12
Price	0.976	1.02
Quality	0.409	2.45
Taste	0.454	2.20
Safety	0.636	1.57
Packaging	0.819	1.22
Nutritional value	0.546	1.83
Beef cattle breed	0.659	1.52
Mean VIF	—	1.56

**Table 5:** Current status of brand awareness across demographic groups.

Characteristic	Group	N	Mean	SD	Test Statistic	*p*-Value
Brand awareness	Overall	273	2.88	0.89	—	—
Age	18–25 years	83	2.66	0.80	χ^2^ = 8.32	0.080
	26–35 years	37	2.84	1.07		
	36–45 years	74	2.99	0.97		
	46–55 years	54	2.94	0.74		
	>55 years	25	3.16	0.90		
Gender	Male	78	3.03	0.93	z = 1.73	0.084
	Female	195	2.82	0.88		
Education	Junior high school or below	42	2.76	0.98	χ^2^ = 6.41	0.170
	High school/vocational	73	3.08	0.86		
	Vocational college	25	3.04	0.73		
	Undergraduate	122	2.76	0.89		
	Graduate or above	11	2.82	0.98		
Residence	Rural	50	2.58	0.76	z = −2.45	0.014
	Urban	223	2.94	0.91		
Marital status	Unmarried	109	2.73	0.89	z = −2.22	0.027
	Married	164	2.97	0.89		
Household buyer	No	104	2.67	0.85	z = −3.01	0.003
	Yes	169	3.00	0.90		
Household monthly income	≤CNY 5000	109	2.83	0.86	χ^2^ = 10.67	0.031
	CNY 5001–10,000	113	2.96	0.76		
	CNY 10,001–15,000	31	2.52	1.31		
	CNY 15,001–20,000	10	3.30	0.95		
	>CNY 20,000	10	3.00	0.94		

Note: Mann–Whitney U tests were used for binary groups, and Kruskal–Wallis tests were used for multi-category groups.

**Table 6:** Baseline regression results.

Variable	Model 1				Model 2			
	B	SE	Z	Exp(B)	B	SE	Z	Exp(B)
Price	−0.297 **	0.124	−2.390	0.743	−0.327 **	0.136	−2.410	0.721
Quality	0.382	0.234	1.640	1.465	0.246	0.260	0.950	1.279
Taste	0.148	0.214	0.690	1.159	0.136	0.237	0.570	1.145
Safety	0.181	0.191	0.950	1.199	0.206	0.206	1.000	1.228
Packaging	−0.230	0.145	−1.590	0.794	−0.205	0.154	−1.330	0.815
Nutritional value	−0.469 **	0.215	−2.180	0.625	−0.446 *	0.229	−1.950	0.640
Cattle breed	0.300 **	0.150	2.010	1.350	0.294 *	0.162	1.810	1.342
Constant	0.828	0.818	1.010	2.289	−1.602	0.996	−1.610	0.201
Brand awareness	—	—	—	—	1.064 ***	0.201	5.300	2.897
Chi-square	19.040				53.500			
Cox & Snell R^2^	0.067				0.178			
Nagelkerke R^2^	0.098				0.260			

Note: *, **, and *** indicate statistical significance at the 10%, 5%, and 1% levels, respectively.

**Table 7:** Mediation model test results.

Variable	Model 3 (BA → PI)		Model 4 (BA&PI → PB)	
	B	SE	B	SE
Brand awareness	0.134 ***	0.039	1.034 ***	0.210
Constant	2.732 ***	0.217	−4.330 ***	1.333
Purchase intention	—	—	0.978 ***	0.297
Product attributes	Controlled		Controlled	
R^2^/Pseudo R^2^	0.109		0.206	

Note: *** indicates statistical significance at the 1% level. Product attribute variables include price, quality, taste, safety, packaging, nutritional value, and beef cattle breed. Model 3 is estimated by OLS; Model 4 is estimated by binary logit.

**Table 8:** Mediation effect test results.

Effect	Estimate	SE	95% CI
Total effect (BA → PB)	0.178 ***	0.032	[0.116, 0.241]
Direct effect (BA → PB|PI)	0.165 ***	0.032	[0.103, 0.228]
Indirect effect (via PI)	0.021 **	0.010	[0.006, 0.048]

Note: ** and *** indicate statistical significance at the 5% and 1% levels, respectively.Effects are expressed on the probability scale (average marginal effects). The indirect effect equals the product of the BA → PI coefficient and the average marginal effect of PI in the logit model (Hayes and Preacher [39]), tested with 5000 bootstrap resamples. The SE and CI for the total and direct effects are based on the delta method; the SE and CI for the indirect effect are based on 5000 bootstrap resamples with bias correction (BC).

**Table 9:** Effect decomposition of the mediation model.

Method	Effect	Estimate	SE	z/t	95% CI	Mediated Share
KHB (logit scale)	Total	1.165 ***	0.216	5.41	[0.743, 1.588]	100%
	Direct	1.034 ***	0.210	4.93	[0.623, 1.446]	88.8%
	Indirect	0.131 **	0.054	2.41	[0.024, 0.238]	11.2%
LPM (all OLS)	Total	0.166 ***	0.028	5.90	[0.110, 0.221]	100%
	Direct	0.147 ***	0.028	5.20	[0.091, 0.203]	88.7%
	Indirect	0.019 **	—	—	[0.005, 0.042]	11.3%

Note: ** and *** indicate statistical significance at the 5% and 1% levels, respectively. KHB = Karlson–Holm–Breen decomposition (Karlson et al. [40]); LPM = linear probability model. For KHB, the total effect is the reduced-model coefficient of BA, the direct effect is the full-model coefficient, and the indirect effect is their difference; SE, z, and CI are analytic. For LPM, the indirect effect is computed as the product of the BA → PI coefficient and the PI → PB coefficient, with a 5000-resample bias-corrected bootstrap CI (BC). SE and z/t are not reported for the LPM indirect effect because it is obtained via bootstrap rather than an analytic formula.

**Table 10:** Robustness check results.

Variable	Model 5	Model 6	Model 7
	5% Winsorization	Probit Model	Adding Demographics
Brand awareness	1.081 *** (0.203)	0.628 *** (0.114)	1.019 *** (0.214)
Product attributes	Controlled	Controlled	Controlled
Demographics	—	—	Controlled
Observations	273	273	273
Chi-square	52.43	53.95	72.78
Pseudo R^2^	0.166	0.171	0.231

Note: *** indicates statistical significance at the 1% level. Product attribute variables include price, quality, taste, safety, packaging, nutritional value, and beef cattle breed. Demographics include gender, age, education, residence, marital status, household income, and household buyer.

**Table 11:** Heterogeneity analysis results.

Variable	(1) Gender		(2) Residence		(3) Age			(4) Household Income		
	Female	Male	Rural	Urban	Young	Middle-Aged	Older	Low	Middle	High
Brand awareness	1.462 *** (0.273)	0.413 (0.330)	1.882 *** (0.701)	0.872 *** (0.213)	0.517 ** (0.236)	2.431 *** (0.900)	1.244 ** (0.516)	0.838 *** (0.298)	1.642 *** (0.470)	0.729 ** (0.356)
Product attributes	Controlled	Controlled	Controlled	Controlled	Controlled	Controlled	Controlled	Controlled	Controlled	Controlled
Estimator	Logit	Logit	Firth logit	Logit	Logit	Firth logit	Firth logit	Logit	Logit	Firth logit
Observations	195	78	50	223	120	74	79	109	113	51
LR/Wald χ^2^	50.75	13.43	10.78	31.10	10.30	12.47	13.35	18.37	27.58	9.54
Nagelkerke R^2^	0.229	0.145	—	0.130	0.067	—	—	0.137	0.248	—

Note: ** and *** indicate statistical significance at the 5% and 1% levels, respectively. Standard errors are in parentheses. Product attribute variables include price, quality, taste, safety, packaging, nutritional value, and beef cattle breed. Firth’s penalized logistic regression (Firth [41]; Heinze and Schemper [42]) was used for small or separated subgroups; for these models, the reported statistic is the Wald chi-square, and Pseudo R^2^ is not reported.

## Data Availability

The data supporting the findings of this study are not publicly available because the dataset includes personal data from participants who consented under the conditions of confidentiality and non-disclosure, and we are obliged to uphold their privacy rights. However, the data are available from the corresponding author, Y.W.Z., upon reasonable request and with respect to the aforementioned.
